# Niche-specific microbial diversity, interactions, and functional potential within the spinach microbiome

**DOI:** 10.1016/j.crmicr.2025.100475

**Published:** 2025-09-26

**Authors:** Dhivya P. Thenappan, Wisnu Adi Wicaksono, Gabriele Berg, Vijay Joshi

**Affiliations:** a Texas A&M AgriLife Research and Extension Center, Uvalde, TX, 78801, USA; b Institute of Environmental Biotechnology, Graz University of Technology, Graz, 8010, Austria; c Leibniz Institute for Agricultural Engineering and Bioeconomy (ATB), Potsdam, 14469, Germany; d Institute for Biochemistry and Biology, University of Potsdam, Potsdam, 14476, Germany; e Department of Horticultural Sciences, Texas A&M University, College Station, TX, 77843, USA

**Keywords:** Spinach, Rhizosphere, Leaf episphere, Root endosphere, Amplicon sequencing

## Abstract

Understanding the spatial organization and functional diversity of microbiomes across plant compartments is essential for unraveling the complex interactions between plants and their associated microbial consortia. This study examined the microbial communities associated with spinach (*Spinacia oleracea* L.) across five ecological niches, using 16S rRNA and ITS amplicon sequencing, in two commercial conventional cultivars: Hammerhead and Traverse. Microbial diversity, community composition, co-occurrence networks, and functional potential showed cultivar-specific effects, especially in the fungal community. The niche was a significant factor influencing all microbiome parameters, showing the same pattern in both cultivars: highest in bulk soil, followed by the rhizosphere, leaf episphere, and finally the root and leaf endosphere. We observed clear niche differentiation and enrichment of niche-specific genera. A core microbiome was identified, comprising 10 bacterial and 6 fungal taxa, with *Streptomyces, Bacillus*, and *Rubrobacter* being the key bacterial genera, and *Alternaria* and *Cladosporium* being the dominant fungi. SourceTracker2 analysis revealed a limited contribution of bulk soil to the rhizosphere (∼25 %), while the rhizosphere and leaf episphere were the primary sources for endosphere communities. Fungal communities showed higher transfer rates (75–96 %) between niches compared to bacterial communities (19–93 %). Co-occurrence network analysis revealed that Traverse had a denser microbial network than Hammerhead, with key hubs such as *Streptomycetaceae* and *Chaetomiaceae.* Inferred functional potential suggested metabolic capabilities in microbial communities across spinach niches, including those of potential plant pathogens. The microbial spatial distribution and identification of spinach from this study lays the foundation for designing targeted strategies to mitigate pathogen risks and introduce beneficial microbial functions into crops.

## Highlights

• Amplicon sequencing analyzed bacterial and fungal microbiomes in spinach niches.
• Microbial diversity varied by niche, highest in bulk soil and roots enriched in *Streptomyces*.
• Compartments showed niche-specific core and enriched microbial taxa.
• Co-occurrence networks revealed positive interactions supporting plant health.
• Functional predictions indicated niche-driven traits and limited bulk soil-phyllosphere links.

## Graphical abstract

**Figure:**
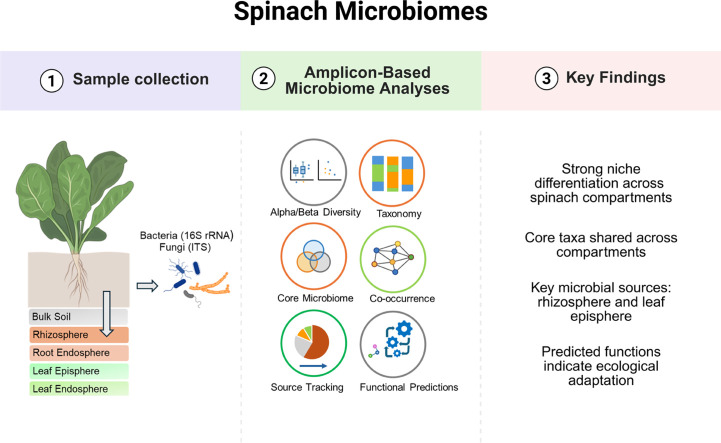

## 1 Introduction

Plant-associated microbial communities inhabit diverse habitats, including seeds, the rhizosphere, and the phyllosphere (Cordovez et al., 2019; Abdelfattah et al., 2023). These microbiomes consist of both potential beneficial and pathogenic organisms that coexist in niche-specific communities, influencing plant health and development (Trivedi et al., 2020). Plant–microbiota interactions contribute to a broader plant phenotype shaped by microbial activity, plant physiology, and environmental stimuli. Understanding these interactions is essential for predicting and managing plant diseases, enhancing crop yields, and identifying phenotypic outcomes modulated by microbial associations (Singer et al., 2019). Significantly, these interactions are often cultivar-dependent, as different plant genotypes attract distinct microbiome members that confer resistance to abiotic and biotic stresses or promote growth and nutrition (Berg and Smalla, 2009). Therefore, investigating the impact of environmental factors and host genetic variation on the plasticity of plant–microbiome interactions is crucial.

Plant-microbe interactions and co-evolution have shaped the diversity of plants, as well as their associated microbiota, over the long term (Delaux and Schornack, 2021). This process was continued by man-made domestication and breeding (Cardinale et al., 2015; Abdelfattah et al., 2022). The specific, mainly beneficial part of the plant microbiota is vertically transmitted and complemented by the acquisition of site-specific microorganisms, allowing local adaptation (Bergna et al., 2018). In addition, the composition of the microbiome is significantly influenced by plant niches; for example, in soybeans, both spatial and temporal dynamics have been shown to independently shape microbial communities across compartments, with niche specializations selecting for only well-adapted taxa (Moroenyane et al., 2021). Along the soil–root continuum, host selection increases as microbial diversity decreases and plant-associated microbiomes become more specialized from the bulk soil to the root interior (Reinhold-Hurek et al., 2015). Phyllosphere communities of field-grown annual crops, such as spinach, are more diverse than stable root-associated microbiomes due to exposure to fluctuating environmental and biotic factors, including climate, insect activity, and plant surface characteristics (Liu et al., 2020).

Spinach (*Spinacia oleracea* L.) is a vital leafy vegetable, mainly produced in China, the US, Turkey, and Japan. China leads with 91 % of global output, or 27.52 million tons annually. The US produces about 0.44 million tons, primarily from California, Arizona, New Jersey, and Texas (FAOSTAT, 2019; USDA-NASS, 2019). Spinach is a nutrient-rich leafy green packed with vitamins, minerals, and antioxidants, offering health benefits such as improved blood sugar control, reduced cancer risk, and enhanced overall nutrition (Bhattarai and Shi, 2021).

Consuming fresh vegetables increases gut microbiota diversity, potentially introducing beneficial genes, such as those involved in vitamin and short-chain fatty acid production, which promotes gut stability, immunity, and overall health (Stephen et al., 2023; Wicaksono et al., 2023). However, raw vegetable consumption, particularly, leafy greens, poses food safety risks due to contamination by pathogens like Shiga toxin-producing *Escherichia coli* (STEC), *Salmonella* spp., *Shigella* spp., *Yersinia* spp., and *Listeria monocytogenes*, leading to the USFDA's 2020 Leafy Greens STEC Action Plan (Mogren et al., 2018; FDA, 2020). The plant phyllosphere, particularly in spinach, is a hotspot for antibiotic-resistant bacteria (ARB) and antibiotic resistance genes (ARGs), with mobile genetic elements (MGEs) facilitating the spread of resistance (Sun et al., 2021; Yin et al., 2022). Despite its agricultural importance, research on spinach microbiomes remains limited, and comparative studies on how different cultivars shape microbiome composition are lacking. This study focuses particularly on spinach microbiomes in Texas, where no prior reports on microbiomes associated with commercial cultivars exist. We hypothesized that the niche would be the primary driver of microbial community composition and function, but that cultivar-level effects may also influence core taxa and functional potential. Two commercial baby spinach cultivars, 'Traverse' (semi-savoy) and 'Hammerhead' (full-savoy), were selected based on their contrasting leaf morphologies. These differences may influence microhabitat conditions and the formation of distinct microbial communities, particularly in the phyllosphere. The specific objectives of this study were to: (1) investigate the diversity, composition, and structure of bacterial and fungal microbiomes across five plant niches in both cultivars (2) identify niche-specific core and enriched microbial taxa ; and (3) infer co-occurrence patterns and explore the functional potential of microbiomes, to better understand their roles in plant health, productivity, and environmental adaptation.

## 2 Materials and methods

### 2.1 Sampling and processing

Soil and plant samples were collected in October 2023 from a private farm in Zavala County, Texas, USA, from 30-day-old plants of two commercial baby spinach cultivars, Traverse and Hammerhead. The farm cultivated spinach on the same plot for over 2 years under a conventional farming system. Fertilization included the application of urea (62.58 kg N/ha), ammonium sulfate (37.8 kg N/ha), monoammonium phosphate (8.5 kg P₂O₅/ha), potash (15.12 kg K₂O/ha), MicroMerge® 5144 (3.4 kg/ha), and elemental zinc (1.81 kg/ha), followed by a humic acid-rich amendment, Resurge (4.54 kg/ha).

Bulk soil samples were collected using an auger to extract soil cores from around the roots of Traverse and Hammerhead cultivars at a depth of approximately 15–20 cm. Random spinach plants (*n* = 5) were uprooted along the rows and in the beds to collect rhizosphere soil, root, and leaf samples at a depth of approximately 10–20 cm. Excess soil was removed by gently shaking the roots when they were separated. We collected shoot samples (*n* = 5) from the same uprooted plants to study the leaf endosphere and episphere. Each cultivar had three composite samples, consisting of five soil cores, root, and leaf samples collected along its rows, in a portable Styrofoam box with cooling elements. Subsequently, the soil samples were sieved through a 2 mm mesh to remove pebbles, roots, and organic material, and then separated into two sections. Five grams of soil samples were taken and stored at -20 °C until the DNA was extracted for the studies on the microbial community, abundance, and function. Samples from other compartments were processed with modifications based on Kusstatscher et al. (2020).Rhizosphere and leaf episphere samples were homogenized for 1 min in 30 mL of 0.85 % sodium chloride buffer and then transferred to an ultrasonic cleaner, where they were sonicated for 1 min at 40 Hz. (Branson 5200 ultrasonic cleaners, Branson Ultrasonics, CT, USA). After centrifuging the homogenate for 20 min at 4 °C at 3500 g, the resulting pellet was kept at -80 °C until DNA extraction. The roots and leaves from the same plants were rinsed with 70 % ethanol for one minute, followed by three washes in sterile distilled water to process the root and leaf endosphere samples. The samples were kept at -80 °C after being flash-frozen in liquid nitrogen.

### 2.2 DNA extraction, and amplicon sequencing

Total DNA was extracted from 250 mg of each sample using the DNeasy PowerSoil Pro kit (Qiagen Inc., Germantown, MD, USA) according to the manufacturer's instructions. The Denovix DS-11 spectrophotometer was used to assess the quality of the DNA. The extracted DNA was stored at -20 °C until subsequent PCR analysis. To investigate bacterial and fungal diversity, the V5-V7 [799F: 5′-AACMGGATTAGATACCCKG-3′ (Chelius and Triplett, 2001), 1193R: 5′-ACGTCATCCCCACCTTCC-3′ (Bodenhausen et al., 2013)] region of the bacterial 16S ribosomal RNA gene and ITS1 [ITS1–1F: 5′-CTTGGTCATTTAGAGGAAGTAA-3′ (Gardes and Bruns, 1993), ITS1R: 5′-GCTGCGTTCTTCATCGATGC-3′ (White et al., 1990)] of the fungal ribosomal internal transcribed spacer region were amplified. The amplicons were sequenced at Novogene Corporation (Novogene Corporation Inc., USA) using Illumina NovaSeq 2 × 300 bp paired-end sequencing. The raw reads were deposited in the NCBI Sequence Read Archive under Bioproject accession numbers PRJNA1189603 (Amplicon sequencing of spinach-associated bacteria communities) and PRJNA1189602 (Amplicon sequencing of spinach-associated fungal communities).

### 2.3 Bioinformatics

Reads that were paired-end were quality-checked and demultiplexed using cutadapt (Martin, 2011). The open-source QIIME2 version 2024.2.0 pipeline (https://qiime2.org) was employed to conduct additional analysis of the demultiplexed reads (Bolyen et al., 2019). After removing primer sequences, the DADA2 algorithm in QIIME2 was used to quality filter, denoise, and eliminate chimeric sequences (Callahan et al., 2016) to produce a feature table and representative sequences known as amplicon sequence variations (ASVs). Taxonomic classification was conducted using the VSEARCH classifier, with the SILVA v138 and UNITE v10 databases serving as reference databases for bacteria and fungi, respectively (Rognes et al., 2016; Quast et al., 2013; Nilsson et al., 2019). For further statistical analysis, plant-derived chloroplast, mitochondrial, and non-target sequences were removed. Using PICRUSt2 and the default analysis parameters, functional predictions of the bacterial community were carried out (Douglas et al., 2020). The tools and algorithms that PICRUSt2 uses include HHMER, EPA-NG, GAPPA, and castor to align ASVs to reference sequences, position them into a reference tree, and perform hidden-state prediction functions, respectively (Barbera et al., 2019, Czech et al., 2020, Douglas et al., 2020, Eddy, 1998, and Louca and Doebeli, 2018). At both the gene level (KEGG orthologs) and pathway level (MetaCyc) (Kanehisa and Goto, 2000; Karp et al., 2002), functional prediction analysis was implemented. The FUNGuild (Nguyen et al., 2016) open annotation tool was used to perform functional analysis on fungal feature tables. It allows parsing fungal community information by ecological guild. Additionally, we utilized the Multiple Bacterial Pathogen Detection (MBPD) pipeline to identify potential pathogens within the microbial community (Yang et al., 2023).

### 2.4 Statistical analyses

The resulting ASV counts, taxonomy table, phylogenetic tree, and experimental metadata were imported into R (version 4.3.1) using RStudio (version 2023.9.0.463) (R Core Team, 2020). The *phyloseq* 1.38.0 (McMurdie and Holmes, 2013) and *vegan* 2.5.7 (Oksanen et al., 2013) packages were then used for processing. The amplicon datasets were subjected to alpha and beta diversity analyses after being rarefied to a minimum sampling depth of 500 reads per sample, as illustrated in Additional File 1: Fig. S1. Alpha rarefaction curves were plotted using the '*ranacapa*' Shiny web application to demonstrate sequencing depth (Kandlikar et al., 2018). The Shannon diversity, InvSimpson, and Pielou's evenness indices were used to calculate microbial alpha diversity. Meanwhile, the non-parametric Kruskal-Wallis test, followed by Dunn's multiple comparisons with the Bonferroni FDR method, was implemented for diversity comparisons between cultivar groups and niches. To investigate the impact of compartment type and cultivar on the composition of microbial communities, beta diversity analyses were performed using permutational analysis of variance (PERMANOVA, 999 permutations) and a normalized Bray-Curtis dissimilarity matrix. Pairwise comparisons were applied to significant factors (and combinations) in *vegan* using the *'pairwise.adonis2*' function, with p-values adjusted using the Bonferroni method. Principal coordinate analysis (PCoA), dendrograms, and non-metric multidimensional scaling (NMDS) were employed to visualize the distance matrices. The microbial taxonomic composition was visualized using stacked bar plots.

The LEfSe (linear discriminant analysis effect size) method, implemented in MicrobiomeAnalyst (Chong et al., 2020; Dhariwal et al., 2017; Segata et al., 2011), was used to identify the microbial genera associated with each sample type. The linear discriminant analysis (LDA) threshold was established at 2 with a p-value cut-off of 0.05. The core microbiome was defined as genera present in ≥90 % of samples per compartment (80 % for the leaf endosphere), with detection thresholds ranging from 0.01 % to 0.9 % relative abundance. To explore microbial interactions, we constructed two co-occurrence networks in R using the '*psych*' package, considering only strong (|r| > 0.60) and statistically significant (*p* < 0.01) correlations. The resulting networks were visualized using Gephi (v0.10.1) (Bastian et al. 2009). We subsequently calculated topological properties, including the average degree, graph density, and the average clustering coefficient (ACC), were subsequently calculated using the '*igraph*' R package (Csardi and Nepusz, 2006). We used the '*SourceTracker2*' package (Knights et al., 2011) was used to assess the proportion of microbiota transferred between spinach niches, estimating the source-sink relationships of microbiomes. A similar analysis was performed for each spinach compartment to identify its role as a source or sink. For functional predictions, DESeq analysis (Love et al., 2014) was performed using the '*DESeq2*' package to compare inferred functions and bacterial abundance between sample types, utilizing non-normalized functions and an ASV abundance table. KEGG orthologs and bacterial ASVs were classified as significantly differentially abundant if the log fold change was >2 and the adjusted *P* value was <0.05, following the Benjamini-Hochberg adjustment. Significantly differentially abundant MetaCyc pathways were identified with log fold change >1 and adjusted *P* value <0.05.

## 3 Results

### 3.1 General assessment of 16S rRNA and ITS amplicon libraries

Amplicon sequencing generated 1,780,477 and 2,827,164 high-quality reads for bacterial and fungal communities, respectively. Bacterial sequences were processed using paired-end reads, whereas fungal sequences were processed with single-end reads due to poor paired-end data quality. Following the removal of non-target reads (such as mitochondrial, chloroplast, and unassigned reads), 14,027 bacterial and 1074 fungal amplicon sequence variations (ASVs) were identified. Following subsampling to 500 reads per sample, 2100 bacterial and 351 fungal ASVs remained. As sequencing depth increased, rarefaction curves (Fig. S1A, B) showed a plateau in species richness across all five niches, indicating that sufficient sequencing depth had been achieved.

### 3.2 Niches influence microbial diversity, while cultivars show limited impact

**Fig. 1:**
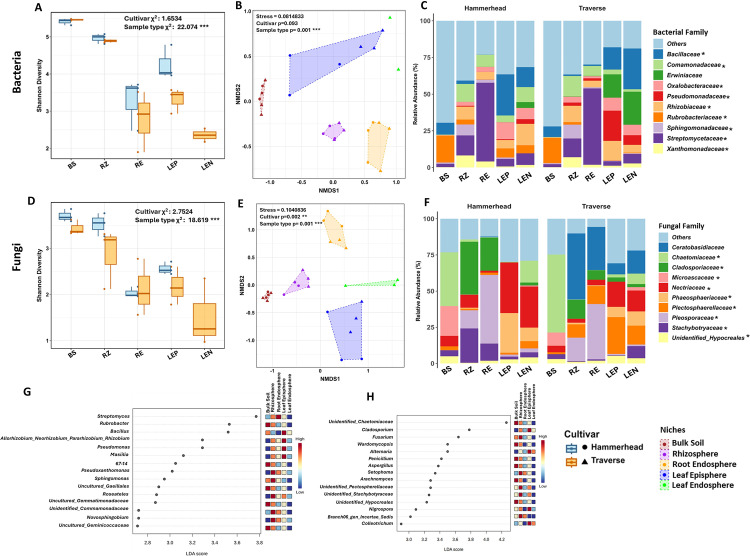
Microbial community patterns across spinach cultivar niches. Shannon diversity of bacterial (A) and fungal (D) communities across niches; asterisks indicate significant differences (*P ≤ 0.05; **P ≤ 0.01; ***P ≤ 0.001). NMDS plots of beta diversity based on Bray–Curtis dissimilarity for bacteria (B) and fungi (E); significance tested by PERMANOVA. Relative abundances of dominant bacterial (C) and fungal (F) families; asterisks indicate LEfSe-significant differences (*P* ≤ 0.05). LEfSe dot plots of differentially abundant bacterial (G) and fungal (H) genera (LDA > 2.6, FDR-adjusted *P* ≤ 0.05); bar color denotes relative abundance (blue: low, red: high). BS: Bulk soil; RZ: Rhizosphere; RE: Root endosphere; LEP: Leaf episphere; LEN: Leaf endosphere.

Bacterial and fungal diversity varied across ecological niches. Alpha diversity analyses showed that ecological niches, but not cultivar, significantly influenced diversity, explaining 22.0 % of bacterial and 18.7 % of fungal variation, while cultivar had no significant effect (Fig. 1A,D). Bulk soil had the highest microbial diversity (bacterial H':5.4; fungal H': 3.7). Diversity followed the order: bulk soil > rhizosphere > leaf episphere > root endosphere > leaf endosphere (Kruskal-Wallis and ANOVA, *P* < 0.05). This pattern was consistent with InvSimpson metrics, with only minor variation in the fungal component. Pielou's evenness increased gradually in both above- and belowground niches (Fig. 1A, D; Fig. S1C-F; Table S1).

Beta diversity analyses indicated that both, ecological niches and cultivars influenced microbial community structure, although cultivar effects were limited and significant only for fungal communities. PERMANOVA showed that cultivars accounted for 3.9 % of bacterial and 7.6 % of fungal variation, while niches explained 46.3 % and 48.1 %, respectively. NMDS revealed clear clustering by niche, with significant bacterial and fungal variation in the rhizosphere and leaf episphere across cultivars (Fig. 1B, E). PCoA indicated that principal components for niche explained 40.2 % of bacterial and 46.7 % of fungal variation (Fig. S2A, B). Hierarchical clustering further supported stronger niche-based than cultivar-based clustering (Fig. S2C, D).

### 3.3 Taxonomic composition of spinach microbiome

Taxonomic analysis of ASVs across all niches identified 32 bacterial phyla and 363 families, along with 8 fungal phyla and 120 families. Among bacterial phyla, *Proteobacteria* (42.4 %) were the most dominant, followed by *Actinobacteriota* (37.9 %) and *Firmicutes* (12.3 %). *Bacteroidota, Chloroflexi, Gemmatimonadota,* and *Myxococcota* each comprised approximately 1.4 % of the total bacterial community. Fungal communities were primarily composed of *Ascomycota* (88.1 %) and *Basidiomycota* (11.5 %), with *Mortierellomycota* and *Chytridiomycota* contributing <0.5 %. Though no significant cultivar differences were observed, *Basidiomycota* was more abundant in root and shoot compartments in Traverse (Fig. S3 A, B; Table S2).At the family level, the ten most prominent microbial families were identified, with the remaining taxa grouped as "others." The proportion of bacterial "others" was higher belowground (45.3 %) than in the phyllosphere (26.2 %), indicating greater diversity in soil and rhizosphere. *Streptomycetaceae* was the most abundant bacterial family (15.9 %), especially in the root endosphere, followed by *Bacillaceae, Rhizobiaceae, Comamonadaceae, and Rubrobacteriaceae. Erwiniaceae, Oxalobacteraceae, and Pseudomonadaceae* were specific to the phyllosphere, while *Sphingomonadaceae* occurred in both the rhizosphere and phyllosphere (Fig. 1C). Among fungi, *Nectriaceae* and *Pleosporaceae* were most abundant (12.2 %), with *Nectriaceae* predominant in the phyllosphere and *Pleosporaceae* in the root endosphere. *Chaetomiaceae* and *Ceratobasidiaceae* were also prevalent, with *Ceratobasidiaceae* unique to Traverse. *Microascaceae, Ceratobasidiaceae, Cladosporiaceae*, and *Stachybotryaceae* were more commonly found in soil and the rhizosphere. The phyllosphere had a higher proportion of fungal 'others' (27.9 %) than belowground compartments (15.1 %) (Fig. 1F; Table S3). Collectively, key bacterial and fungal families accounted for significant compositional differences between the belowground and phyllosphere microbiomes.

Linear discriminant analysis Effect size (LEfSe) analysis identified bacterial and fungal genera differentially enriched across spinach compartments, with no cultivar-specific differences (LDA  >  3.0, *P*  <  0.05; Fig. 1G,H). Among bacteria, *Rubrobacter* dominated the bulk soil, while *Pseudoxanthomonas* and *Streptomyces* were enriched in the rhizosphere and root endosphere, respectively. Notably, *Streptomyces* gradually increased from the soil to the rhizosphere to the root endosphere. *Bacillus, Allorhizobium-Neorhizobium-Pararhizobium-Rhizobium*, and *Masilia* were enriched in the leaf episphere, and *Bacillus* was moderately abundant in the leaf endosphere. For fungi, unidentified_*Chaetomiaceae* were enriched in bulk soil, while *Fusarium* and *Setophoma* were more abundant in the rhizosphere and root endosphere, respectively, and *Cladosporium* in the phyllosphere.

Analysis of ASVs linked to potential pathogens identified 9 bacterial and 7 fungal ASVs, mainly in the leaf episphere and endosphere, with lower levels in root and soil compartments. Traverse exhibited higher bacterial ASV abundance, notably *Pantoea vagans* (27.5 %) and *P. agglomerans* (10.6 %), while Hammerhead contained *Bacillus mycoides* (15.8 % in leaf episphere, 6.8 % in leaf endosphere) (Fig. S4A; Table S8). *Streptomyces turgidiscabies*, associated with plant pathogenicity, was detected in the roots and leaves of Traverse. Fungal ASVs with potential pathogenic members were restricted to the leaf episphere: Traverse contained Ustilaginaceae (25 %), and Hammerhead had *Anthracocystis* (35.8 %), *Erysiphaceae* (28.1 %), and *Arthrographis*. A few ASVs could be assigned to the species level with high confidence, including *Sporisorium trachypogonis-spicati* and *Arthrographis kalrae*, which are known to include pathogenic representatives (Fig. S4A; Table S4). Overall, Hammerhead had higher fungal ASV abundance in leaves, in contrast to the bacterial patterns observed in Traverse.

### 3.4 Defining the core microbiome of spinach niches

**Fig. 2:**
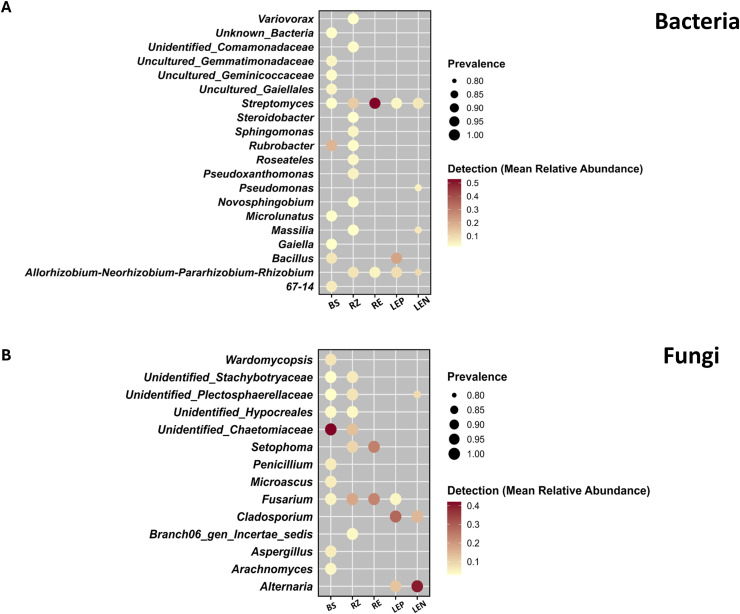
Dot plot showing the core bacterial (A) and fungal genera (B) of spinach-associated niches under commercial farming systems. Core taxa was identified based on prevalence with a detection threshold >0. 01 % across all samples. BS: Bulk soil; RZ: Rhizosphere; RE: Root endosphere; LEP: Leaf episphere; LEN: Leaf endosphere.

Core microbiome analysis identified 10 bacterial and 6 fungal taxa, which, while representing a small fraction of ASVs, accounted for substantial read abundance (bacteria: 6.1 % of reads, 0.07 % ASVs; fungi: 17.7 % of reads, 0.56 % ASVs). *Rubrobacter* dominated bulk soil (18 %), *Bacillus* was prevalent in the leaf episphere (22 %), and *Streptomyces* prevailed in the root endosphere (53 %). The *Allorhizobium–Neorhizobium–Pararhizobium–Rhizobium* group was detected across multiple niches and was abundant in the phyllosphere (∼9 %). For fungi, unidentified_*Chaetomiaceae* dominated bulk soil (42 %), *Setophoma* was enriched in the root endosphere (24 %), *Alternaria* dominated the leaf endosphere (40 %), and *Cladosporium* was abundant in leaf niches (∼28 %). *Fusarium* was consistently present across compartments (5–23 %). These results demonstrate compartment-specific enrichment of core bacterial and fungal taxa (Fig. 2; Table S5).

### 3.5 Tracking microbial transfer between niches in spinach

**Fig. 3:**
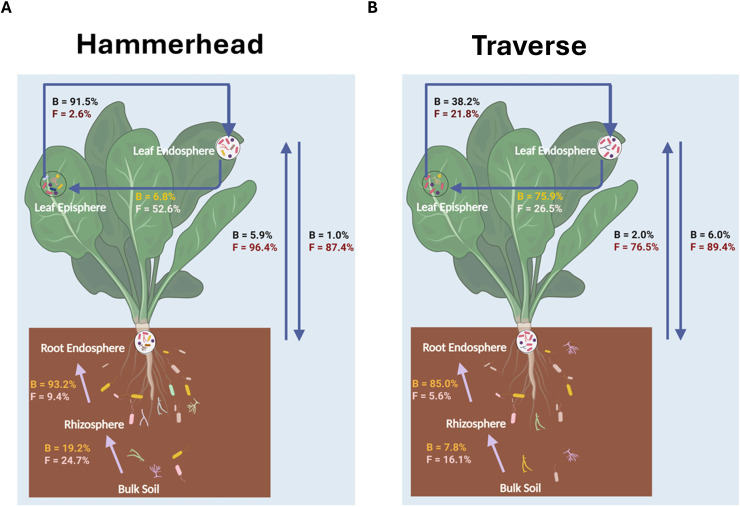
Percentage of bacteria and fungi in niches associated with source types based on SourceTracker2 analysis. Cultivars Hammerhead (A) and Traverse (B) are shown separately. *B* = Bacteria; *F* = Fungi.

SourceTracker2 analysis indicated minimal direct microbiome transfer from soil to the phyllosphere via the rhizosphere. Substantial transfer was observed from bulk soil to rhizosphere, from rhizosphere to root endosphere, and within the phyllosphere from leaf episphere to leaf endosphere. Fungal transfer between root and leaf endospheres was high (75–96 %), while bacterial transfer within belowground and phyllosphere compartments (19–93 %) was higher than that of fungi. Root and leaf endospheres shared the largest transfer of fungal communities. Cultivar differences were minor: Hammerhead showed slightly greater soil microbiota enrichment belowground, while Traverse exhibited higher phyllosphere transfer (Fig. 3).

### 3.6 Co-occurrence patterns in bacterial and fungal communities

**Fig. 4:**
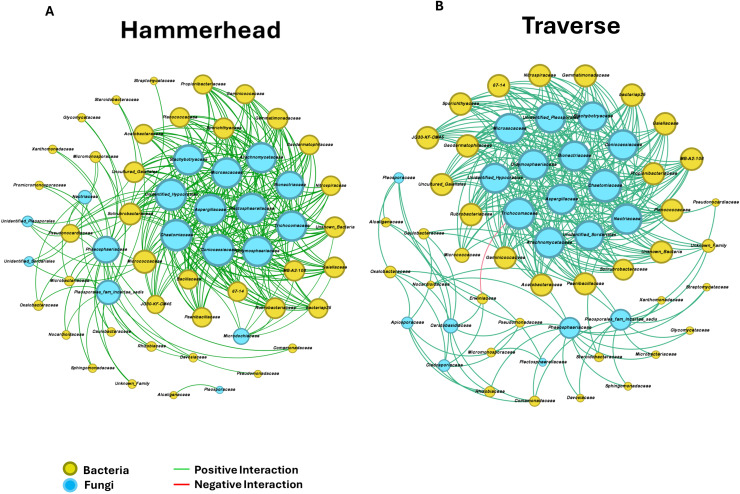
Co-occurrence network analysis of bacteria - fungi communities in cultivars Hammerhead (A) and Traverse (B). The size of each node is proportional to the number of connections. The connections in the network represent statistical significance (|r| > 0.5, *p* < 0.05). Green and red lines indicate negative and positive interactions, respectively, between families.

Network analysis of bacterial-fungal interactions revealed 56 nodes and 277 edges in Hammerhead and 57 nodes and 295 edges in Traverse, with positive correlations in 100 % and 99.7 % of edges, respectively. In Hammerhead, networks were primarily connected through *Micrococcaceae* and *Solirubrobacteraceae*, while Traverse was dominated by *Acetobacteraceae, Gaillaceae*, and *Geminicoccaceae. Chaetomiaceae* was the most connected fungal family in both cultivars. Only Traverse showed negative correlations between *Erwiniaceae* and *Trichomaceae* (Fig. 4). Bacterial-bacterial subnetworks included 40 nodes and 844 edges in Hammerhead and 30 nodes and 248 edges in Traverse, with 100 % and 99.2 % positive correlations, respectively. Fungal-fungal subnetworks comprised 18 nodes and 184 edges in Hammerhead and 21 nodes and 153 edges in Traverse, both with 100 % positive correlations. Hammerhead exhibited higher average degrees and graph density in both bacterial and fungal subnetworks, indicating greater network complexity. In Traverse, *Nitrospiraceae* and *Enterobacteraceae* displayed negative correlations (Fig. S5; Table S6).

### 3.7 Microbial functional potential inferred from PICRUSt2 and FUNGuild

PICRUSt2 predicted 432 bacterial metabolic pathways from 16S rRNA gene data, with 231 showing significant variations between compartments (*P*  ≤  0.05). NSTI values ranged from 0.043 to 0.27, indicating reliable prediction quality (Li et al., 2024). Core pathways, including aerobic respiration, branched-chain amino acid biosynthesis, the TCA cycle, the pentose phosphate pathway, and fatty acid metabolism, were consistently abundant across all compartments in both cultivars. Gondoate biosynthesis was detected only in bulk soil, rhizosphere, and phyllosphere of Traverse (Fig. S6).

**Fig. 5:**
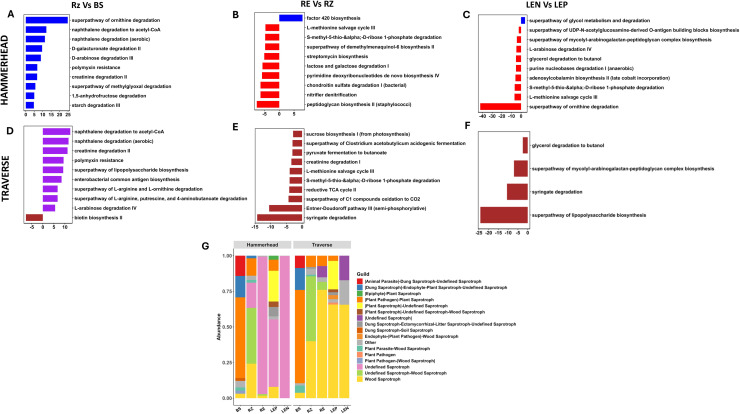
Functional profiling of bacterial and fungal communities in spinach niches and cultivars. Differential enrichment analysis of bacterial functional pathways predicted by PICRUSt2 (A) and compared across niches: Rhizosphere vs. Bulk Soil (RZ vs. BS), Root Endosphere vs. Rhizosphere (RE vs. RZ), and Leaf Endosphere vs. Leaf Episphere (LEN vs. LEP) using DESeq2 (adjusted *P* < 0.05). Diversity and composition of fungal functional groups (D) at the guild level across cultivars, predicted by FUNGuild.

Differential abundance analysis (padj < 0.001) revealed compartment-specific enrichment across three transitions: bulk soil vs. rhizosphere, rhizosphere vs. root endosphere, and leaf episphere vs. leaf endosphere. Traverse rhizospheres were enriched in lipopolysaccharide biosynthesis and enterobacterial antigens, while Hammerhead showed higher ornithine degradation. Root endospheres of Traverse exhibited higher predicted siderophore and antioxidant pathways, whereas Hammerhead was enriched in cofactor metabolism (factor 420). Leaf endospheres showed cultivar-specific differences in energy, cofactor, and amino acid catabolism pathways (Fig. 5A-F; Table S7).

Fungal communities were assigned to predicted trophic modes and guilds using FUNGuild, with no significant differences observed between cultivars or compartments (*P* > 0.05). Bulk soil was dominated by plant pathogen–plant saprotroph fungi (78 %), while saprotrophs were more prevalent in root and leaf compartments. At the guild level, undefined saprotrophs (17.7–99.8 %) dominated Hammerhead endospheres, whereas wood saprotrophs (39.9–76 %) were more abundant in Traverse. Minor cultivar differences were also noted in the leaf episphere (Fig. 5G; Table S8).

## 4 Discussion

In the present study, ecological niche was the primary factor shaping microbial diversity across spinach compartments, with alpha diversity decreasing from bulk soil to internal plant tissues in both cultivars. This pattern suggests host filtering and niche specialization along the soil–plant continuum.

Bacterial diversity was primarily shaped by niche, with no significant effect from cultivar, suggesting stable bacterial communities across genotypes. In contrast, fungal diversity was influenced by both niche and cultivar. PERMANOVA showed that cultivars explained 7.6 % of the variation in the fungal community (*p* < 0.05), indicating a limited host genotype effect. The sampling design, with five plants per sample and three replicates per cultivar per niche, may have limited detection of subtle cultivar effects. Full-savoy cultivars such as Hammerhead have highly crinkled leaves with greater surface area, more ridges, stomata, and glandular trichomes, creating additional microhabitats and promoting nutrient accumulation. Semi-savoy cultivars like Traverse provide fewer colonization sites (Morelock and Correll, 2008; Lopez-Velasco et al., 2011). These morphological differences may explain the modest cultivar-specific fungal differences observed. Additionally, conventional fertilization, including nitrogen, phosphorus, potassium, micronutrients, and humic acid, likely influenced spinach microbiomes. Nitrogen can favor copiotrophic bacteria and reduce the protective capacity of resident communities, while phosphorus and potassium impact nutrient cycling and community composition. Humic acid amendments are known to enrich microbial diversity and taxa associated with plant defense (Delitte et al., 2021; Lumactud et al., 2022). These inputs may have shaped the observed community patterns in the rhizosphere and phyllosphere, highlighting the importance of management practices in influencing crop-associated microbiomes.

Based on these diversity patterns, spinach-associated microbial communities included both broadly distributed and niche-enriched taxonomic groups. Across compartments, *Actinobacteriota* (e.g., *Rubrobacteraceae, Streptomycetaceae*), *Proteobacteria* (e.g., *Comamonadaceae, Rhizobiaceae, Sphingomonadaceae, Oxalobacteraceae, Pseudomonadaceae, Erwiniaceae, Xanthomonadaceae*), and *Firmicutes* (*Bacillaceae*) were abundant, consistent with findings from other leafy greens (Dees et al., 2015; Williams et al., 2013). In contrast, *Acidobacteriota* and *Chloroflexi* were less prevalent in the phyllosphere, as previously reported (Lopez-Velasco et al., 2013). *Streptomyces* dominated the root endosphere, consistent with its enrichment in root-associated niches of other plants (Worsley et al., 2020; Viaene et al., 2016), while enrichment of the *Allorhizobium–Neorhizobium–Pararhizobium–Rhizobium* complex in the leaf endosphere has been reported to confer stress-tolerance traits (Fan et al., 2023). Fungal communities also displayed niche specificity: *Ceratobasidiaceae* (*Basidiomycota*), detected only in Traverse, includes both symbiotic and pathogenic species; *Phaeosphaeriaceae* and *Pleosporaceae* (*Ascomycota*) dominated above- and belowground environments, respectively, reflecting associations with mildew and root or postharvest diseases (Veldre et al., 2013; Steenwerth et al., 2021). *Penicillium*, found in the root endosphere, is a known postharvest pathogen and has been reported on spinach (Dukare et al., 2022; Gu et al., 2025), although its role in leafy greens is unclear. *Alternaria*, enriched in leaf niches, includes *A. alternata*, the causal agent of spinach leaf spot (Gilardi et al., 2019), indicating a potential disease risk.

At the species level, 16S amplicon analysis identified *Pantoea vagans, Pantoea agglomerans, Bacillus mycoides*, and *Streptomyces turgidiscabies* as potentially pathogenic. However, confirmation at the strain level will require culture-based or virulence gene-based validation. Most fungal ASVs were identified at the genus level, with limited reliable species-level assignments which should therefore be interpreted with caution. Traverse had more bacterial pathogens, while Hammerhead showed a greater abundance of fungal pathogens, especially in the leaf episphere, potentially influencing disease susceptibility under intensive horticultural practices (Berg et al., 2017; Brennan et al., 2022). We hypothesize that Hammerhead’s crinkled leaf surface may increase moisture retention and spore deposition, contributing to this enrichment. Nonetheless, functional prediction tools such as FUNGuild could not assign functions to much of the fungal community, which points to the need for improved database coverage (Nguyen et al., 2016). Taken together, these findings emphasize the importance of microbiome monitoring in leafy vegetables to protect crop and consumer health.

Moreover, our study identified core microbiomes across bulk soil, rhizosphere, and root endosphere (*Streptomyces, Rubrobacter, Allorhizobium-Neorhizobium-Pararhizobium-Rhizobium, Fusarium, Aspergillus*, and *Penicillium*), suggesting selective recruitment from soil and possible internal root-to-leaf transmission (Lugtenberg and Kamilova, 2009; Hardoim et al., 2015). Regarding microbial transfer between spinach niches, though bulk soil served as the primary reservoir of rhizosphere microbiota (Hu et al., 2020), only ∼20 % of rhizosphere communities were bulk soil-derived. This finding highlights the potential role of seed-associated microbes in early rhizosphere assembly (Garrido-Sanz and Keel, 2025). Rhizosphere microbial composition is also influenced by soil type, plant genotype, and root exudates. Overall, microbial transfer from soil to the phyllosphere via the rhizosphere was minimal. Yet, the observed overlap between leaf and root communities suggests the possibility of internal plant transmission routes.

Network analysis identified distinct differences between the two cultivars, with correlation analysis showing predominantly positive bacterial-fungal interactions in both. Hammerhead, with its highly crinkled leaves, supported a more complex microbial co-occurrence network, likely due to greater structural diversity on its leaf surfaces. This complexity may reflect denser microbial interactions in heterogeneous habitats rather than increased community stability. Although, Hammerhead is reported to be resistant to white rust (Morelock and Correll, 2008; Spawton et al., 2024); whether its network structure contributes to this resistance remains untested. In contrast, Traverse, a semi-savoy cultivar with smoother leaf architecture, supported a simpler microbial network and exhibited negative associations between *Erwinaceae* (potential pathogens) and *Trichomaceae* (antagonistic fungi), suggesting possible biocontrol interactions. Both abundant and rare taxa contributed to network connectivity; for example, less common *Solirubrobacteraceae*, involved in nutrient cycling and plant defense (Özbolat et al., 2023; Paina et al., 2024), were highly connected, whereas abundant *Streptomycetaceae* had minimal connectivity. These results suggest that leaf morphology influences microbial network complexity and co-occurrence patterns, with both abundant and rare taxa potentially playing important ecological roles. Further research is needed to confirm these relationships, considering data compositionality and environmental factors (Matchado et al., 2021).

Predicted bacterial functions suggested that core metabolic processes, including carbon metabolism, respiration, and amino acid biosynthesis, were maintained across spinach compartments, while niche-specific differences reflected local adaptations. For example, rhizosphere communities were enriched in xenobiotic degradation pathways, whereas leaf and root endospheres exhibited functions that could be linked to microbial stress tolerance and nutrient interactions, such as siderophore, antioxidant, and cofactor metabolism. Cultivar-level differences were less pronounced than compartment effects, with Traverse enriched in pathways supporting host colonization and Hammerhead enriched in pathways promoting microbial survival. Fungal guild predictions demonstrated functional similarity across niches and cultivars, with bulk soil acting as a potential reservoir for both beneficial and potentially pathogenic taxa. Overall, these patterns suggest that microbial communities may maintain core functions despite taxonomic variation, but amplicon-based predictions are indirect and require validation using metagenomic or transcriptomic approaches.

## 5 Conclusion

In conclusion, this study offers a comprehensive characterization of bacterial and fungal communities in spinach niches from two commercial cultivars, Hammerhead and Traverse. The rhizosphere and bulk soil served as key reservoirs of microbial diversity, with functional predictions revealing niche-specific metabolic traits. Hammerhead, known for its resistance to white rust, showed a more complex and positively connected microbial network, which may indicate greater community stability, though causality cannot be inferred. These insights can help growers and breeders select cultivars with beneficial microbiome profiles and develop strategies to promote beneficial taxa while reducing food safety risks. Future studies incorporating additional cultivars, replicates, agronomic measurements, and meta-omics approaches will be important to confirm these findings and translate microbiome knowledge into practical applications, including direct assessment of pathogenic potential via strain-level or virulence gene-based analyses. Finally, inoculants based on core microbiome members consistently found across niches and cultivars may also provide reliable benefits for plant growth promotion and disease suppression.

## CRediT authorship contribution statement

**Dhivya P. Thenappan:** Conceptualization, Data curation, Formal analysis, Investigation, Methodology, Software, Validation, Visualization, Writing – original draft. **Wisnu Adi Wicaksono:** Data curation, Formal analysis, Software, Validation, Writing – review & editing. **Gabriele Berg:** Writing – review & editing. **Vijay Joshi:** Conceptualization, Funding acquisition, Project administration, Resources, Supervision, Writing – review & editing.

## Declaration of competing interest

The authors declare that they have no known competing financial interests or personal relationships that could have appeared to influence the work reported in this paper.

## Data Availability

NCBI SRABioproject accession numbers PRJNA1189603 (Amplicon sequencing of spinach-associated bacteria communities) and PRJNA1189602 (Amplicon sequencing of spinach-associated fungal communities). (Original data)
NCBI SRABioproject accession numbers PRJNA1189603 (Amplicon sequencing of spinach-associated bacteria communities) and PRJNA1189602 (Amplicon sequencing of spinach-associated fungal communities). (Original data)

## Appendix Supplementary materials

**Fig. S1:** Rarefaction curves for 16S rRNA and ITS gene amplicon libraries of all samples (**A, B**). Box-and-whisker plots visualize the InvSimpson (and Pielou Evenness) indices of bacteria (C, E) and fungi (D, **F**) across different niches. Image, image 1

**Fig. S2:** Beta diversity analyses shown as Principal coordinate analysis (PCOA) for bacterial (**A**) and fungal (**B**) communities across different niches in cultivars. Hierarchical clustering of bacteria (**C**) and fungi (**D**) across different niches in cultivars based on Bray-Curtis dissimilarity matrix. Image, image 2

**Fig. S3:** Relative abundances of the dominant bacterial (**A**) and fungal (**B**) phyla across different niches in cultivars. Image, image 3

**Fig. S4:** Bacterial (**A**) and fungal (**B**) amplicon sequence variants (ASVs) with a mean relative abundance over 0.2 % in Spinach niches with identified pathogenic traits. Image, application 4

**Fig. S5:** Co-occurrence Network analyses of bacteria (**A, B**) and fungi (**C, D**) communities in cultivars Hammerhead (**A, C**) and Traverse (**B, D**). The size of each node is proportional to the number of connections it has. The thickness of each connection (edge) between two nodes (yellow) is proportional to the value of Spearman's correlation coefficient (r), and the links represent statistical significance (|r| > 0.6, *p* < 0.01). Green and red lines indicate negative and positive interactions, respectively, between families. Image, image 5

**Fig. S6:** PICRUSt2-based prediction of microbial functional potential across spinach cultivars. The Nearest Sequenced Taxon Index (NSTI) scores for individual samples indicate prediction accuracy (**A**). Relative abundances of predicted functional pathways across niches in cultivars Hammerhead (**B**) and Traverse (**C**) as inferred from PICRUSt2 analysis. Image, image 6

**Table S1:** Alpha diversity metrics of bacteria and fungi communities Image, application 7

**Table S2:** Relative abundance analysis results of bacteria at the phyla and family level Image, application 8

**Table S3:** Relative abundance analysis results of fungi at the phyla and family level Image, application 9

**Table S4:** Detection of potential bacterial pathogens using multiple bacterial pathogen detection (MBPD) Image, application 10

**Table S5:** Core Microbiome analysis results of bacteria and fungi across the niches Image, application 11

**Table S6:** Co-occurrence network analysis results of bacteria and fungi at the family level across the cultivars Image, application 12

**Table S7:** PICRUST2-based Weighted NSTI data and DESEQ2 analysis of the given samples Image, application 13

**Table S8:** FUNGuild analysis of fungal trophic modes and Ecological Guilds Image, application 14

## References

[bib0001] Abdelfattah A., Tack A.J., Lobato C., Wassermann B., Berg G. (2023). From seed to seed: the role of microbial inheritance in the assembly of the plant microbiome. Trends Microbiol..

[bib0002] Abdelfattah A., Tack A.J., Wasserman B., Liu J., Berg G., Norelli J., Wisniewski M. (2022). Evidence for host–microbiome co-evolution in apple. New Phytol..

[bib0003] Barbera P., Kozlov A.M., Czech L., Morel B., Darriba D., Flouri T., Stamatakis A. (2019). EPA-ng: massively parallel evolutionary placement of genetic sequences. Syst. Biol..

[bib0004] Bastian M., Heymann S., Jacomy M. (2009). Proceedings of the international AAAI conference on web and social media.

[bib0005] Berg G., Köberl M., Rybakova D., Müller H., Grosch R., Smalla K. (2017). Plant microbial diversity is suggested as the key to future biocontrol and health trends. FEMS Microbiol. Ecol..

[bib0006] Bergna A., Cernava T., Rändler M., Grosch R., Zachow C., Berg G. (2018). Tomato seeds preferably transmit plant beneficial endophytes. Phytobiomes J..

[bib0007] Berg G., Smalla K. (2009). Plant species and soil type cooperatively shape the structure and function of microbial communities in the rhizosphere. FEMS Microbiol. Ecol..

[bib0008] Bhattarai G., Shi A. (2021). Research advances and prospects of spinach breeding, genetics, and genomics. Veg. Res..

[bib0009] Bodenhausen N., Horton M.W., Bergelson J. (2013). Bacterial communities associated with the leaves and the roots of *Arabidopsis thaliana*. PLoS. One.

[bib0010] Bolyen E., Rideout J.R., Dillon M.R., Bokulich N.A., Abnet C.C., Al-Ghalith G.A. (2019). Reproducible, interactive, scalable and extensible microbiome data science using QIIME 2. Nat. Biotechnol..

[bib0011] Brennan F.P., Alsanius B.W., Allende A., Burgess C.M., Moreira H., Johannessen G.S., Holden N.J. (2022). Harnessing agricultural microbiomes for human pathogen control. ISME Commun..

[bib0012] Callahan B.J., McMurdie P.J., Rosen M.J., Han A.W., Johnson A.J.A., Holmes S.P. (2016). DADA2: high-resolution sample inference from Illumina amplicon data. Nat. Methods.

[bib0013] Cardinale M., Grube M., Erlacher A., Quehenberger J., Berg G. (2015). Bacterial networks and co-occurrence relationships in the lettuce root microbiota. Environ. Microbiol..

[bib0014] Chelius M.K., Triplett E.W. (2001). The diversity of archaea and bacteria in association with the roots of *Zea mays* L. Microb. Ecol..

[bib0015] Chong J., Liu P., Zhou G., Xia J. (2020). Using MicrobiomeAnalyst for comprehensive statistical, functional, and meta-analysis of microbiome data. Nat. Protoc..

[bib0016] Cordovez V., Dini-Andreote F., Carrión V.J., Raaijmakers J.M. (2019). Ecology and evolution of plant microbiomes. Annu. Rev. Microbiol..

[bib0017] Csardi G., Nepusz T. (2006). The igraph software. Complex Syst..

[bib0018] Czech L., Barbera P., Stamatakis A. (2020). Genesis and gappa: processing, analyzing and visualizing phylogenetic (placement) data. Bioinformatics.

[bib0019] Dees M.W., Lysøe E., Nordskog B., Brurberg M.B. (2015). Bacterial communities associated with surfaces of leafy greens: shift in composition and decrease in richness over time. Appl. Environ. Microbiol..

[bib0020] Delaux P.M., Schornack S. (2021). Plant evolution driven by interactions with symbiotic and pathogenic microbes. Science.

[bib0021] Delitte M., Caulier S., Bragard C., Desoignies N. (2021). Plant microbiota beyond farming practices: a review. Front. Sustain. Food Syst..

[bib0022] Dhariwal A., Chong J., Habib S., King I.L., Agellon L.B., Xia J. (2017). MicrobiomeAnalyst: a web-based tool for comprehensive statistical, visual and meta-analysis of microbiome data. Nucleic. Acids. Res..

[bib0023] Douglas G.M., Maffei V.J., Zaneveld J.R., Yurgel S.N., Brown J.R., Taylor C.M., Langille M.G. (2020). PICRUSt2 for prediction of metagenome functions. Nat. Biotechnol..

[bib0024] Dukare A.S., Singh R.K., Jangra R.K., Bhushan B. (2022). Non-fungicides-based promising technologies for managing post-production penicillium induced spoilage in horticultural commodities: a comprehensive review. Food Rev. Int..

[bib0025] Eddy S.R. (1998). Profile hidden Markov models. Bioinformatic.

[bib0026] Fan D., Schwinghamer T., Liu S., Xia O., Ge C., Chen Q., Smith D.L. (2023). Characterization of endophytic bacteriome diversity and associated beneficial bacteria inhabiting a macrophyte. *Eichhornia crassipes*. Front. Plant Sci..

[bib0027] FAOSTAT F. (2019). Food and Agriculture Organization of the United Nations-Statistic Division. https://www.fao.org/faostat/en/#data.

[bib0028] FDA U. (2020). Leafy greens STEC action plan. https://www.fda.gov/food/foodborne-pathogens/2020-leafy-greens-stec-action-plan.

[bib0029] Gardes M., Bruns T.D. (1993). ITS primers with enhanced specificity for basidiomycetes—application to the identification of mycorrhizae and rusts. Mol. Ecol..

[bib0030] Garrido-Sanz D., Keel C. (2025). Seed-borne bacteria drive wheat rhizosphere microbiome assembly via niche partitioning and facilitation. Nat. Microbiol..

[bib0031] Gilardi G., Matic S., Gullino M.L., Garibaldi A. (2019). First report of *Alternaria alternata* causing leaf spot on spinach (*Spinacia oleracea*) in Italy. Plant Dis..

[bib0032] Gu G., Zhou B., Yang Y., Nou X., Millner P.D., Zhang B., Luo Y. (2025). Microbial profiles of commercially packaged baby spinach from hydroponic controlled environment agriculture and soil-based open field production. Food Control.

[bib0033] Hardoim P.R., van Overbeek L.S., Berg G., Pirttilä A.M., Compant S., Campisano A., Döring M., Sessitsch A. (2015). The hidden world within plants: ecological and evolutionary considerations for defining functioning of microbial endophytes. Microbiol. Mol. Biol. Rev..

[bib0034] Hu Q., Tan L., Gu S., Xiao Y., Xiong X., Zeng W.-a. (2020). Network analysis infers the wilt pathogen invasion associated with non-detrimental bacteria. NPJ Biofilms Microb..

[bib0035] Kandlikar G.S., Gold Z.J., Cowen M.C., Meyer R.S., Freise A.C., Kraft N.J.B., Moberg-Parker J., Sprague J., Kushner D.J., Curd E.E. (2018). ranacapa: an R package and shiny web app to explore environmental DNA data with exploratory statistics and interactive visualizations. F1000Res..

[bib0036] Kanehisa M., Goto S. (2000). KEGG: kyoto encyclopedia of genes and genomes. Nucleic. Acids. Res..

[bib0037] Karp P.D., Riley M., Paley S.M., Pellegrini-Toole A. (2002). The metacyc database. Nucleic. Acids. Res..

[bib0038] Knights D., Kuczynski J., Charlson E.S., Zaneveld J., Mozer M.C., Collman R.G. (2011). Bayesian community-wide culture-independent microbial source tracking. Nat. Methods..

[bib0039] Kusstatscher P., Wicaksono W.A., Thenappan D.P., Adam E., Müller H., Berg G. (2020). Microbiome management by biological and chemical treatments in maize is linked to plant health. Microorganisms.

[bib0040] Li L., Kuzyakov Y., Xu Q., Guo H., Zhu C., Guo J., Ling N. (2024). Bacterial communities in cropland soils: taxonomy and functions. Plant Soil.

[bib0041] Liu H., Brettell L.E., Singh B. (2020). Linking the phyllosphere microbiome to plant health. Trends. Plant Sci.

[bib0042] Lopez-Velasco G., Carder P.A., Welbaum G.E., Ponder M.A. (2013). Diversity of the spinach (*Spinacia oleracea*) spermosphere and phyllosphere bacterial communities. FEMS Microbiol. Lett..

[bib0043] Lopez-Velasco G., Welbaum G.E., Falkinham J.O., Ponder M.A. (2011). Phyllopshere bacterial community structure of spinach (*Spinacia oleracea*) as affected by cultivar and environmental conditions at time of harvest. Diversity.

[bib0044] Louca S., Doebeli M. (2018). Efficient comparative phylogenetics on large trees. Bioinformatics.

[bib0045] Love M.I., Huber W., Anders S. (2014). Moderated estimation of fold change and dispersion for RNA-seq data with DESeq2. Genome Biol..

[bib0046] Lumactud R.A., Gorim L.Y., Thilakarathna M.S. (2022). Impacts of humic-based products on the microbial community structure and functions toward sustainable agriculture. Front. Sustain. Food Syst..

[bib0047] Lugtenberg B., Kamilova F. (2009). Plant-growth-promoting rhizobacteria. Annu. Rev. Microbiol..

[bib0048] Martin M. (2011). Cutadapt removes adapter sequences from high-throughput sequencing reads. EMBnet. J..

[bib0049] Matchado M.S., Lauber M., Reitmeier S., Kacprowski T., Baumbach J., Haller D., List M. (2021). Network analysis methods for studying microbial communities: a mini review. Comput. Struct. Biotechnol. J..

[bib0050] McMurdie P.J., Holmes S. (2013). phyloseq: an R package for reproducible interactive analysis and graphics of microbiome census data. PLoS. One.

[bib0051] Mogren L., Windstam S., Boqvist S., Vågsholm I., Söderqvist K., Rosberg A.K., Alsanius B. (2018). The hurdle approach–a holistic concept for controlling food safety risks associated with pathogenic bacterial contamination of leafy green vegetables. A review. Front. Microbiol..

[bib0052] Morelock T.E., Correll J.C., Prohens J., Nuez F. (2008). Vegetables, I: Asteraceae, Brassicaceae, Chenopodiaceae, and Cucurbitaceae.

[bib0053] Moroenyane I., Tremblay J., Yergeau É. (2021). Temporal and spatial interactions modulate the soybean microbiome. FEMS Microbiol. Ecol..

[bib0054] NASS U. (2019). https://www.nass.usda.gov.

[bib0055] Nguyen N.H., Song Z., Bates S.T., Branco S., Tedersoo L., Menke J., Kennedy P.G. (2016). FUNGuild: an open annotation tool for parsing fungal community datasets by ecological guild. Fungal. Ecol..

[bib0056] Nilsson R.H., Larsson K.-H., Taylor A.F.S., Bengtsson-Palme J., Jeppesen T.S., Schigel D. (2019). The UNITE database for molecular identification of fungi: handling dark taxa and parallel taxonomic classifications. Nucleic. Acids. Res..

[bib0057] Oksanen J., Blanchet F.G., Kindt R., Legendre P., Minchin P.R., O'hara R.B., Oksanen M.J. (2013). Package 'vegan'. Commun. Ecol. Package, Version.

[bib0058] Özbolat O., Sánchez-Navarro V., Zornoza R., Egea-Cortines M., Cuartero J., Ros M., Martínez-Mena M. (2023). Long-term adoption of reduced tillage and green manure improves soil physicochemical properties and increases the abundance of beneficial bacteria in a Mediterranean rainfed almond orchard. Geoderma.

[bib0059] Paina C., Fois M., Asp T., Jensen J., Hansen P.B., Rohde P.D. (2024). The soil microbiome of *Lolium perenne* L. depends on host genotype, is modified by nitrogen level and varies across season. Sci. Rep..

[bib0060] Quast C., Pruesse E., Yilmaz P., Gerken J., Schweer T., Yarza P. (2013). The SILVA ribosomal RNA gene database project: improved data processing and web-based tools. Nucleic. Acids. Res..

[bib0061] Reinhold-Hurek B., Bünger W., Burbano C.S., Sabale M., Hurek T. (2015). Roots shaping their microbiome: global hotspots for microbial activity. Annu. Rev. Phytopathol..

[bib0062] Rognes T., Flouri T., Nichols B., Quince C., Mahé F. (2016). VSEARCH: a versatile open source tool for metagenomics. Peer J..

[bib0063] Segata N., Izard J., Waldron L., Gevers D., Miropolsky L., Garrett W.S., Huttenhower C. (2011). Metagenomic biomarker discovery and explanation. Genome Biol..

[bib0064] Singer E., Bonnette J., Kenaley S.C., Woyke T., Juenger T.E. (2019). Plant compartment and genetic variation drive microbiome composition in switchgrass roots. Environ. Microbiol. Rep..

[bib0065] Spawton K.A., Stein L.A., du Toit L.J. (2024). Evaluation of spinach cultivars for resistance to stemphylium leaf spot (*Stemphylium vesicarium*) and white rust (*Albugo occidentalis*). HortScience.

[bib0066] Steenwerth K.L., Morelan I., Stahel R., Figueroa-Balderas R., Cantu D., Lee J. (2021). Fungal and bacterial communities of 'Pinot noir' must: effects of vintage, growing region, climate, and basic must chemistry. PeerJ..

[bib0067] Stephen J., Manoharan D., Radhakrishnan M. (2023). Immune boosting functional components of natural foods and its health benefits. Food Prod. Process Nutr..

[bib0068] Sun Y., Snow D., Walia H., Li X. (2021). Transmission routes of the microbiome and resistome from manure to soil and lettuce. Environ. Sci. Technol..

[bib0069] Team R.C. (2020). https://www.r-project.org.

[bib0070] Trivedi P., Leach J.E., Tringe S.G., Sa T., Singh B.K. (2020). Plant-microbiome interactions: from community assembly to plant health. Nat. Rev. Microbiol..

[bib0071] Veldre V., Abarenkov K., Bahram M., Martos F., Selosse M.A., Tamm H., Tedersoo L. (2013). Evolution of nutritional modes of ceratobasidiaceae (Cantharellales, Basidiomycota) as revealed from publicly available ITS sequences. Fungal. Ecol..

[bib0072] Viaene T., Langendries S., Beirinckx S., Maes M., Goormachtig S. (2016). *Streptomyces* as a plant's best friend?. FEMS Microbiol. Ecol..

[bib0073] White T.J., Bruns T.L., Taylor J.W., Innis M.A., Gelfand D.H., Snisky J.J., White T.J. (1990). PCR protocols, a Guide to Molecular Methods and Applications.

[bib0074] Wicaksono W.A., Cernava T., Wassermann B., Abdelfattah A., Soto-Giron M.J., Toledo G.V., Berg G. (2023). The edible plant microbiome: evidence for the occurrence of fruit and vegetable bacteria in the human gut. Gut. Microbes.

[bib0075] Williams T.R., Moyne A.L., Harris L.J., Marco M.L. (2013). Season, irrigation, leaf age, and *Escherichia coli* inoculation influence the bacterial diversity in the lettuce phyllosphere. PLoS. One.

[bib0076] Worsley S.F., Newitt J., Rassbach J., Batey S.F., Holmes N.A., Murrell J.C., Hutchings M.I. (2020). *Streptomyces* endophytes promote host health and enhance growth across plant species. Appl. Environ. Microbiol..

[bib0077] Yang X., Jiang G., Zhang Y., Wang N., Zhang Y., Wang X., Wei Z. (2023). MBPD: a multiple bacterial pathogen detection pipeline for One Health practices. Imeta.

[bib0078] Yin Y., Zhu D., Yang G., Su J., Duan G. (2022). Diverse antibiotic resistance genes and potential pathogens inhabit in the phyllosphere of fresh vegetables. Sci. Total. Environ..

