# Supplementary figures and images for: Niche-specific microbial diversity, interactions, and functional potential within the spinach microbiome
Source: Curr Res Microb Sci. 2025 Sep 26;9:100475. doi: 10.1016/j.crmicr.2025.100475 (PMC12538574; doi:10.1016/j.crmicr.2025.100475)

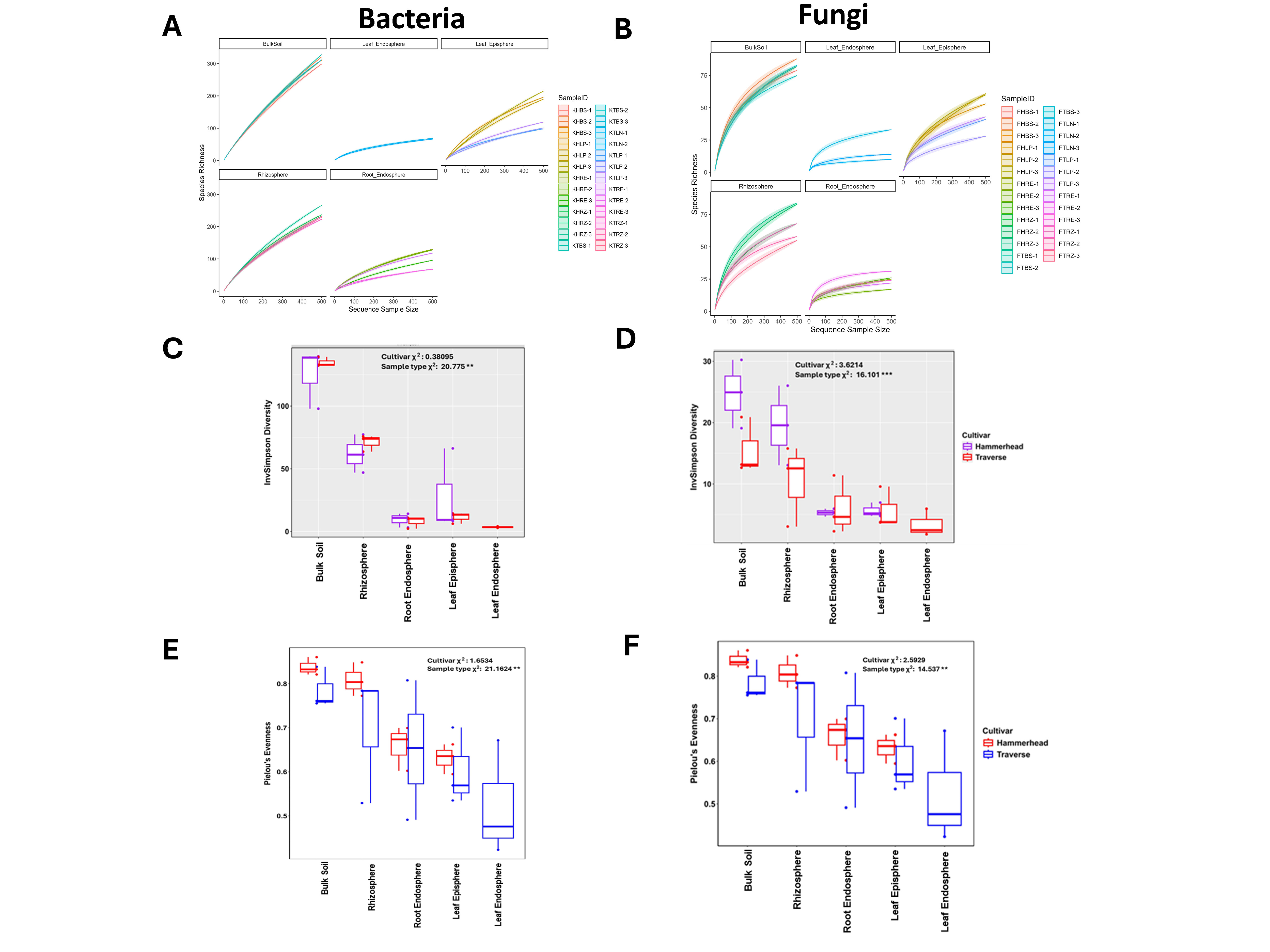

Supplement: Supplementary file 1 — Fig. S1: Rarefaction curves for 16S rRNA and ITS gene amplicon libraries of all samples (A, B). Box-and-whisker plots visualize the InvSimpson (and Pielou Evenness) indices of bacteria (C, E) and fungi (D, F) across different niches. [file mmc1.jpg]

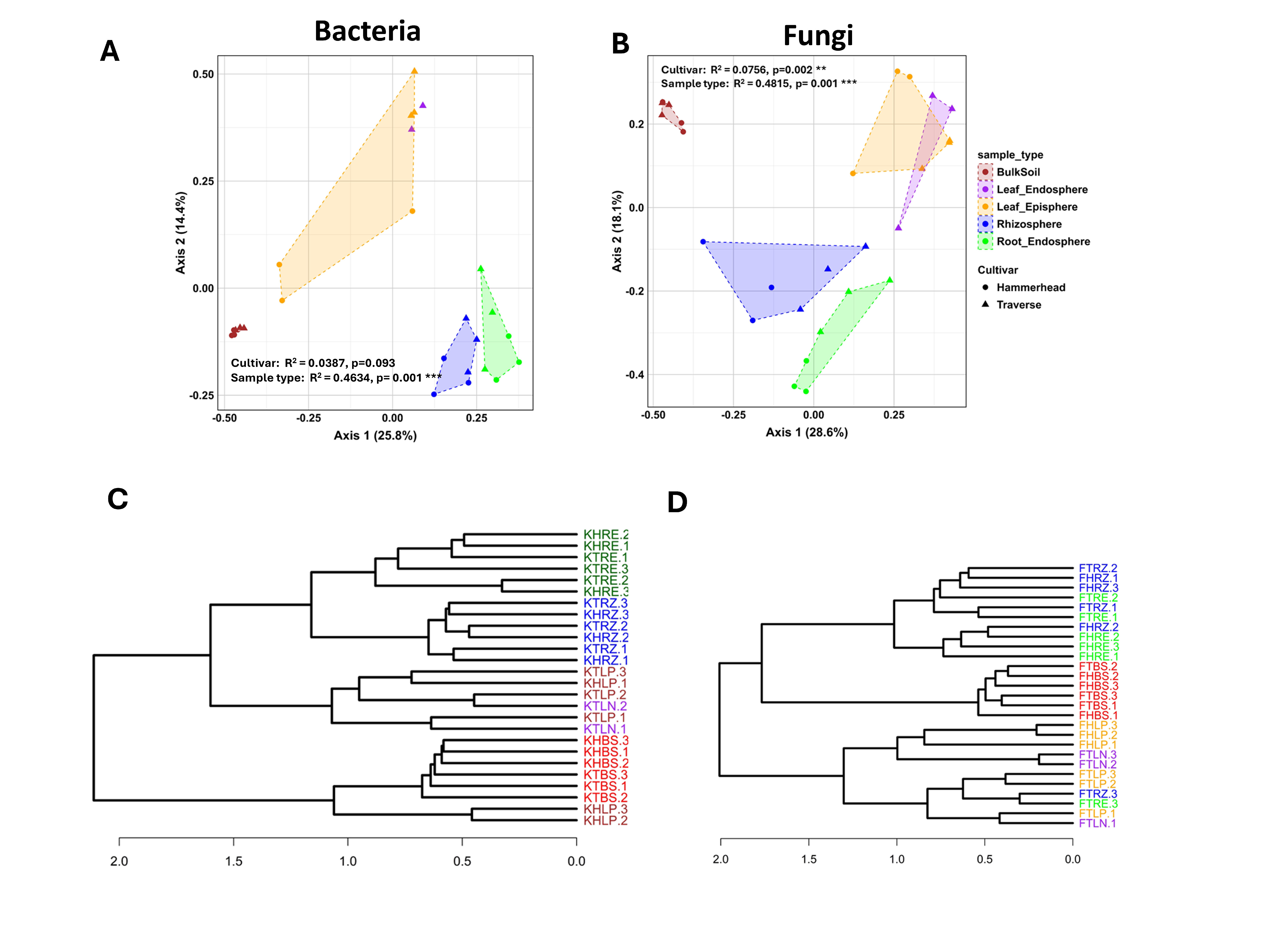

Supplement: Supplementary file 2 — Fig. S2: Beta diversity analyses shown as Principal coordinate analysis (PCOA) for bacterial (A) and fungal (B) communities across different niches in cultivars. Hierarchical clustering of bacteria (C) and fungi (D) across different niches in cultivars based on Bray-Curtis dissimilarity matrix. [file mmc2.jpg]

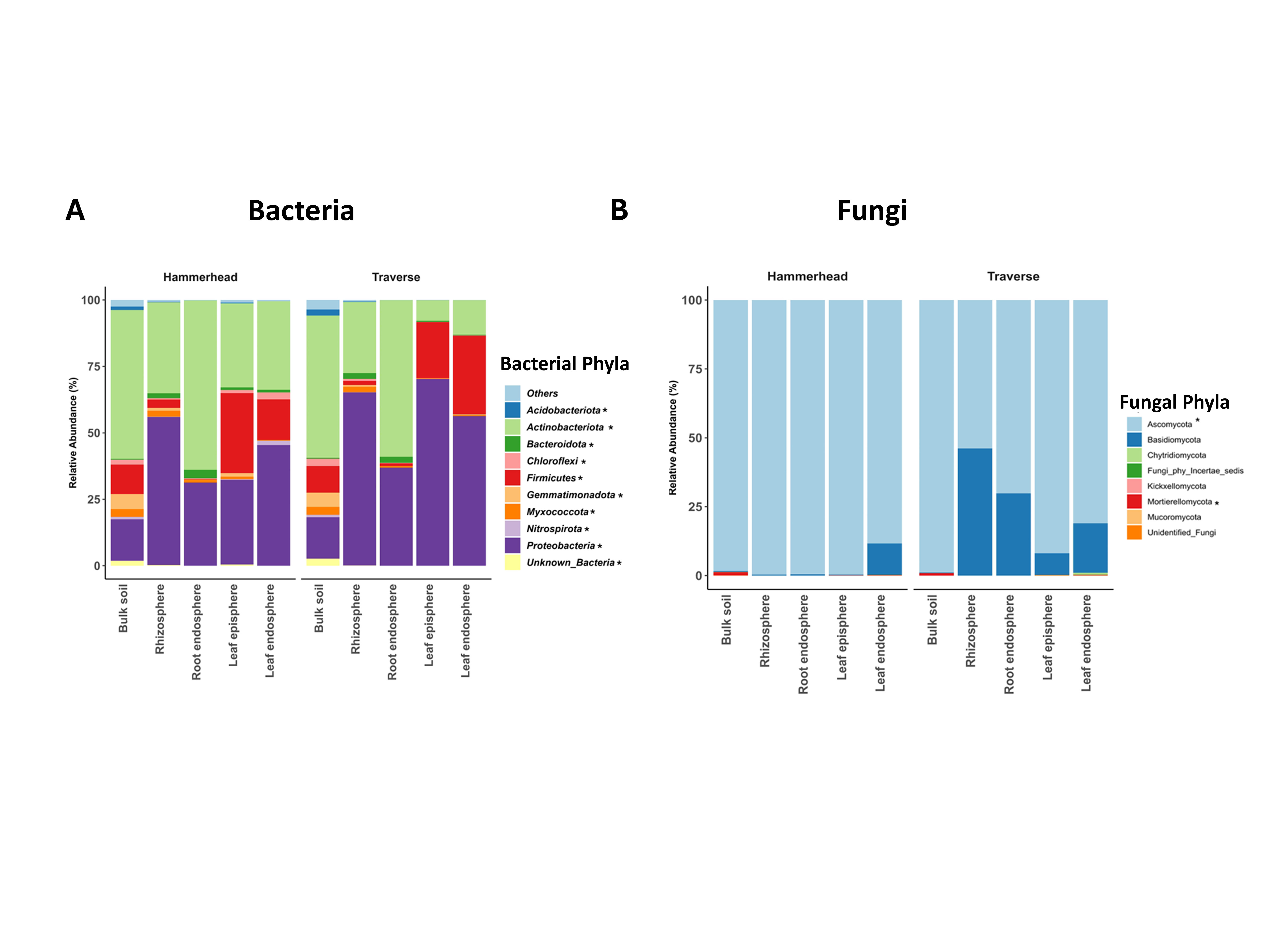

Supplement: Supplementary file 3 — Fig. S3: Relative abundances of the dominant bacterial (A) and fungal (B) phyla across different niches in cultivars. [file mmc3.jpg]

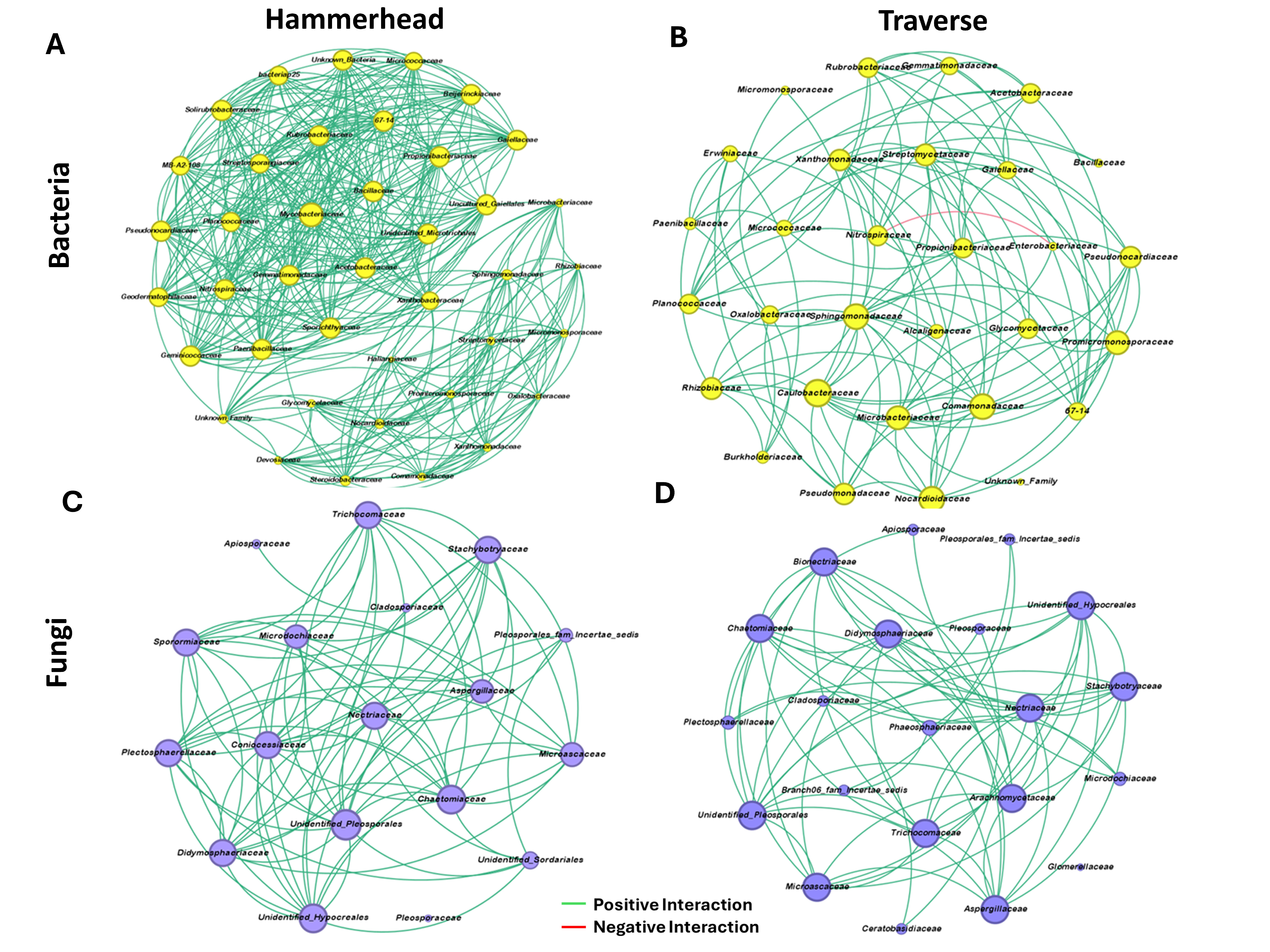

Supplement: Supplementary file 5 — Fig. S5: Co-occurrence Network analyses of bacteria (A, B) and fungi (C, D) communities in cultivars Hammerhead (A, C) and Traverse (B, D). The size of each node is proportional to the number of connections it has. The thickness of each connection (edge) between two nodes (yellow) is proportional to the value of Spearman's correlation coefficient (r), and the links represent statistical significance (|r| > 0.6, p < 0.01). Green and red lines indicate negative and positive interactions, respectively, between families. [file mmc5.jpg]

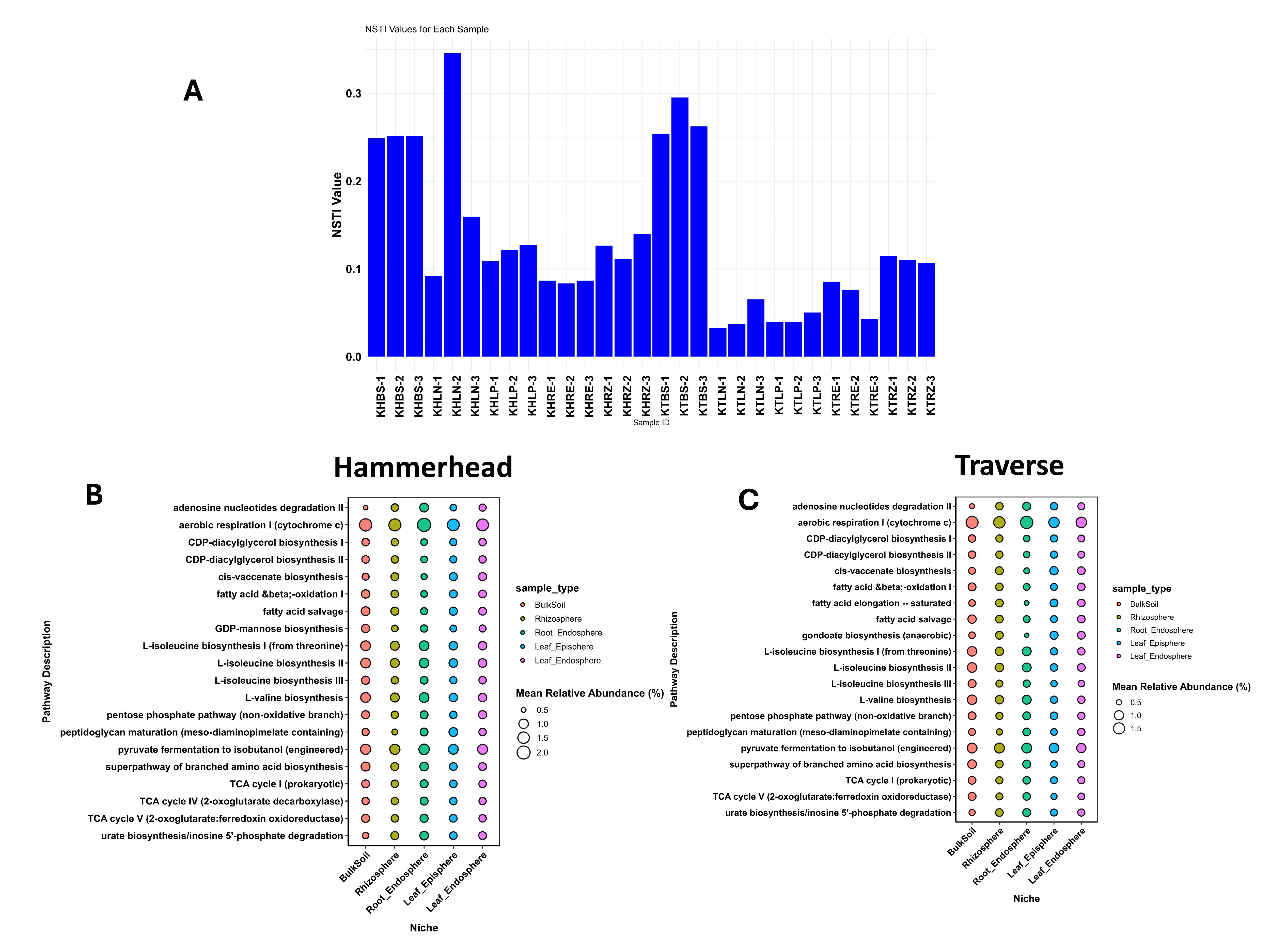

Supplement: Supplementary file 6 — Fig. S6: PICRUSt2-based prediction of microbial functional potential across spinach cultivars. The Nearest Sequenced Taxon Index (NSTI) scores for individual samples indicate prediction accuracy (A). Relative abundances of predicted functional pathways across niches in cultivars Hammerhead (B) and Traverse (C) as inferred from PICRUSt2 analysis. [file mmc6.jpg]
